# A Novel You Only Listen Once (YOLO) Deep Learning Model for Automatic Prominent Bowel Sounds Detection: Feasibility Study in Healthy Subjects

**DOI:** 10.3390/s25154735

**Published:** 2025-07-31

**Authors:** Rohan Kalahasty, Gayathri Yerrapragada, Jieun Lee, Keerthy Gopalakrishnan, Avneet Kaur, Pratyusha Muddaloor, Divyanshi Sood, Charmy Parikh, Jay Gohri, Gianeshwaree Alias Rachna Panjwani, Naghmeh Asadimanesh, Rabiah Aslam Ansari, Swetha Rapolu, Poonguzhali Elangovan, Shiva Sankari Karuppiah, Vijaya M. Dasari, Scott A. Helgeson, Venkata S. Akshintala, Shivaram P. Arunachalam

**Affiliations:** 1 Digital Engineering & Artificial Intelligence Laboratory (DEAL), Mayo Clinic, Jacksonville, FL 32224, USAjay.gohri26@gmail.com (J.G.); rachnakukreja7@gmail.com (G.A.R.P.); helgeson.scott@mayo.edu (S.A.H.); 2 Department of Internal Medicine, Wright Medical Center, Scranton, PA 18503, USA; 3 Department of Internal Medicine, MedStar Union Memorial Hospital, Baltimore, MD 21218, USA; 4 Department of Internal Medicine, Lower Bucks Hospital, Bristol, PA 19007, USA; 5 Department of Internal Medicine, UCHealth Parkview Medical Center, Pueblo, CO 81003, USA; 6 Department of Internal Medicine, Mercy Catholic Medical Center, Darby, PA 19023, USA; charmyparikh18@gmail.com; 7 North Texas Gastroenterology, Denton, TX 76201, USA; 8 Department of Critical Care Medicine, Mayo Clinic, Jacksonville, FL 32224, USA; 9 Division of Pulmonary Medicine, Department of Medicine, Mayo Clinic, Jacksonville, FL 32224, USA; 10 Division of Gastroenterology & Hepatology, Department of Medicine, Johns Hopkins School of Medicine, Baltimore, MD 21287, USA

**Keywords:** GI diseases, bowel sounds, phonoenterogram (PEG), You Only Listen Once (YOLO), artificial intelligence, Mel-frequency Cepstral Coefficients (MFCC), CNN

## Abstract

Accurate diagnosis of gastrointestinal (GI) diseases typically requires invasive procedures or imaging studies that pose the risk of various post-procedural complications or involve radiation exposure. Bowel sounds (BSs), though typically described during a GI-focused physical exam, are highly inaccurate and variable, with low clinical value in diagnosis. Interpretation of the acoustic characteristics of BSs, i.e., using a phonoenterogram (PEG), may aid in diagnosing various GI conditions non-invasively. Use of artificial intelligence (AI) and improvements in computational analysis can enhance the use of PEGs in different GI diseases and lead to a non-invasive, cost-effective diagnostic modality that has not been explored before. The purpose of this work was to develop an automated AI model, You Only Listen Once (YOLO), to detect prominent bowel sounds that can enable real-time analysis for future GI disease detection and diagnosis. A total of 110 2-minute PEGs sampled at 44.1 kHz were recorded using the Eko DUO^®^ stethoscope from eight healthy volunteers at two locations, namely, left upper quadrant (LUQ) and right lower quadrant (RLQ) after IRB approval. The datasets were annotated by trained physicians, categorizing BSs as prominent or obscure using version 1.7 of Label Studio Software^®^. Each BS recording was split up into 375 ms segments with 200 ms overlap for real-time BS detection. Each segment was binned based on whether it contained a prominent BS, resulting in a dataset of 36,149 non-prominent segments and 6435 prominent segments. Our dataset was divided into training, validation, and test sets (60/20/20% split). A 1D-CNN augmented transformer was trained to classify these segments via the input of Mel-frequency cepstral coefficients. The developed AI model achieved area under the receiver operating curve (ROC) of 0.92, accuracy of 86.6%, precision of 86.85%, and recall of 86.08%. This shows that the 1D-CNN augmented transformer with Mel-frequency cepstral coefficients achieved creditable performance metrics, signifying the YOLO model’s capability to classify prominent bowel sounds that can be further analyzed for various GI diseases. This proof-of-concept study in healthy volunteers demonstrates that automated BS detection can pave the way for developing more intuitive and efficient AI-PEG devices that can be trained and utilized to diagnose various GI conditions. To ensure the robustness and generalizability of these findings, further investigations encompassing a broader cohort, inclusive of both healthy and disease states are needed.

## 1. Introduction

The sounds produced by the contractions and mixing of gases and liquids in the digestive system are known as bowel sounds and their electrical recording is called a phonoenterogram (PEG). The traditional method of listening to these sounds, known as auscultation, is a fundamental component of physical examinations of the abdomen. It helps in determining the presence of normal bowel activity and aids in diagnosing gastrointestinal disorders. However, this technique tends to be empirical and subjective; its effectiveness largely depends on the physician’s experience, and it presents challenges in precise documentation and reassessment [1]. There are no objective metrics for evaluating bowel sounds, and a notable absence of clinical research underpins discussions about the value of auscultation [1,2]. This gap is highlighted by past research emphasizing the necessity for enhanced training and education as well as a deeper comprehension of the objective acoustic characteristics of bowel sounds, especially their importance in the perioperative phase [3]. Similarly, management challenges are present in conditions like post-operative ileus (POI) and intestinal obstruction. POI is characterized by a disruption of normal bowel movement after surgery, which results in constipation and an inability to tolerate oral food intake [4]. At present, healthcare professionals depend solely on subjective assessments like listening to bowel sounds, measuring abdominal girth, observing signs like passage of flatus, and observing stool patterns [5]. With a global population nearing eight billion, it is safe to assume that almost everyone has experienced and heard bowel sounds (BSs). However, only a small number of people can effectively use these sounds for patient benefit. Bowel sounds are a universal phenomenon and, due to their intuitiveness as a subject of study, have drawn scientific interest since ancient times.

Since bowel sounds are produced by intestinal contractions [6,7] they are a direct reflection of intestinal motor activity, which is notoriously challenging to measure and assess directly due to the invasive nature of current methods [8]. Auscultation of bowel sounds has been a common, cost-effective method for over 150 years to evaluate various abdominal issues [9]. In practical settings, the characterization and evaluation of bowel sounds are often imprecise and not definitive. Consequently, prior research has shown that there is only low to moderate consistency among different observers when it comes to assessing bowel sounds [10]. Additionally, for chronic illnesses like ulcerative colitis (UC) and Crohn’s disease (CD), regular and ongoing monitoring through endoscopy is essential to assess disease severity and inform treatment strategies [11]. As the annual number of endoscopies rises, with reports indicating 22.2 million procedures in the United States in 2021 [12], the strain on the healthcare system intensifies. Additionally, invasive techniques like endoscopies heighten the risk of complications such as infections and perforations for patients [13].

In the past decade, there has been a growing interest in the research community regarding the use of artificial intelligence (AI) in the field of gastroenterology, like computer-aided auscultation (CAA) [14,15]. This interest is particularly focused on employing AI, particularly wireless devices [16,17], to assist in characterizing diseases during medical procedures, with the goal of enhancing diagnostic accuracy and effectiveness. The use of artificial intelligence, particularly through machine learning and deep learning approaches, is increasingly being integrated into gastrointestinal endoscopy. Key advancements have been made in the development of computer-aided detection and diagnosis systems, especially for identifying colorectal polyps [18]. Recent AI systems have shown impressive sensitivity and accuracy, rivaling even expert human endoscopists. Additionally, AI applications have expanded to include the detection of gastrointestinal bleeding, the identification of inflamed areas, and the diagnosis of specific gastrointestinal infections [19]. Studies conducted by research teams have demonstrated the effectiveness of deep learning in enhancing a range of tasks in gastroenterology. These include detecting colonic polyps and analyzing images obtained from wireless capsule endoscopy (WCE) [20,21].

The primary objective of this research is to develop a novel You Only Listen Once (YOLO) deep learning system for real-time detection and classification of bowel sounds using the Eko DUO stethoscope for data collection. The system employs a 1D-CNN augmented transformer to differentiate between prominent and non-prominent bowel sounds, enhancing the analysis of phonoenterographic data. This approach aims to improve gastrointestinal health assessments and support clinical decisions regarding bowel motility and post-anesthesia feeding protocols. By providing a more objective and consistent alternative to the subjective traditional methods of manual auscultation, the system seeks to increase accuracy and efficiency in bowel sound analysis.

## 2. Materials and Methods

### 2.1. Data Collection

**Figure 1:**
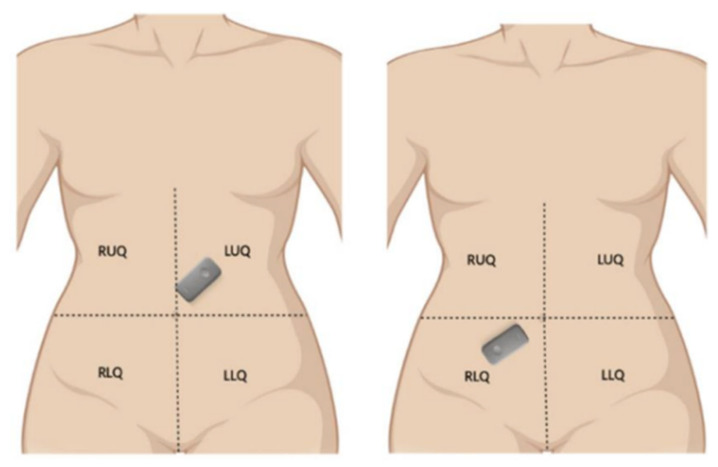
Locations of the Eko DUO stethoscopes during recording sessions.

We utilized the Eko DUO stethoscope to capture bowel sounds from 8 healthy adult volunteers, comprising 5 females and 3 males, under the Mayo Clinic IRB-approved protocol #22-013060. The study specifically targeted two anatomical regions: the left upper quadrant (LUQ) and the right lower quadrant (RLQ), representing the regions around the pyloric sphincter and ileocecal junction, respectively (as referenced in Figure 1).

To ensure consistent sound capture and minimize respiratory motion artifacts, the microphone of the stethoscope was securely affixed using Transpore tape. The tension provided by the tape was optimized to prevent any undue pressure that might influence the gastrointestinal environment. The chosen recording spectrum for the stethoscope’s microphone ranged between 250 and 5000 Hz, conducted in a controlled, noise-minimized environment.

Our data collection involved two specific regimens: Firstly, after an overnight fast, bowel sounds were recorded every 30 min, from 1 h before to 4 h after meal intake. Secondly, we initiated a continuous 24 h monitoring phase, capturing PEGs hourly to observe physiological changes influenced by sleep, meals, activity, and defecation. The entire study yielded a total of 242 min of phonoenterographic data.

For the post-processing phase, we employed Label Studio software for the annotation of bowel sounds. Prior to annotation, recordings were subjected to a 4× amplification. This step was crucial to accurately delineate the onset and end points of prominent bowel sounds. We classified the sounds into two main categories: inaudible/obscure/baseline bowel sounds, which, although not discernible to an unaided ear, became apparent when amplified 16×, and audible/prominent/distinct bowel sounds that were clearly audible without any amplification. It is worth noting that two prominent sounds were considered distinct only if separated by a phase of inaudible or baseline sounds. Lastly, ensuring the reliability of our annotations, the confidence threshold for the observer in distinguishing true bowel sounds from potential background noise was maintained at >85%.

### 2.2. Data Preprocessing

As mentioned previously, the goal of this algorithm is to act as a real-time method for the detection of bowel sounds. Initially, audio data are loaded using the librosa library. This step results in the extraction of both the audio waveform and its corresponding sample rate. Following this, to eliminate potential low-frequency noise that often carries minimal relevance, a high-pass Butterworth filter is applied with a cutoff frequency set at 50 Hz. Subsequent to this filtering, the audio data undergo normalization and are transformed into a 16-bit integer format to facilitate further processing.

Data are then converted into an AudioSegment object, allowing for segmentation. Segmentation pivots on two parameters: a segment_length set at 375 milliseconds and an overlap duration of 200 milliseconds. As the dataset is parsed, intervals featuring prominent sounds are identified. For intervals shorter than the defined segment_length, padding is added, ensuring that the prominent sound is effectively centered within the segment. To address edge cases, where the defined segment may breach the boundaries of the audio file, adjustments are made to the segment’s start or end times, guaranteeing conformity to the audio’s duration. In instances where the interval of prominence exceeds the segment_length, overlapping segments are extracted until the interval’s conclusion. Each of these segments, post-extraction, is archived as a distinct .wav file within a repository earmarked for sounds of prominence.

In parallel, the non-prominent sound intervals are also identified. These intervals are discerned by observing the quietude between successive intervals of prominent sounds. Further, any residual audio data post the final prominent sound are classified as non-prominent segments. The extraction process for these quieter segments mirrors that of their louder counterparts, with each segment being saved in a dedicated directory. At the end of this process, we have a dataset of 6435 prominent sound segments and 36,149 non-prominent sound segments, each of 0.375 s.

### 2.3. Feature Extraction and Augmentation

In the initial stages, our dataset consists of sound files that are read using the librosa library. The Mel-frequency cepstral coefficients (MFCCs) are calculated for each segment, and are used as inputs into a machine learning model. The first step in this process is to convert the time domain signal into the frequency domain using the Short-Time Fourier Transform (STFT).

(1) $$X(k)=\sum_{n=0}^{N-1}\;x(n)\times w(n)\;x\;e^{-j(2\pi /N)\times k\times n}$$

where X(k) is the STFT of x(n), w(n) is the Hamming window, N is the size of the FFT, and k is the frequency index. The frequency spectrum obtained from the STFT is then passed through a series of overlapping triangular filters called the Mel filter bank to get the Mel spectrum. Each filter in the Mel filter bank corresponds to a specific range of frequencies.

The Mel scale is derived from human perception studies, and it reflects the way the human ear perceives frequencies. The conversion between the Hertz scale and the Mel scale is given by

(2) $$m(f)=2595\times log_{10}(1+f/700)$$

where m(f) is the frequency in Mel, and f is the frequency in Hertz. Once we pass the STFT through the Mel filter bank, we take the logarithm of the power at each of the Mel frequencies. This operation accounts for the non-linear human perception of loudness and pitch.

(3) $$L\left(i\right)=log\left(\sum_{k}\;|X\left(k\right){|}^{2}\times H\left(i,k\right)\right)\;$$

where L(i) is the log Mel spectrum for the ith filter. H(i, k) represents the Mel filter bank. Finally, we apply the DCT to the log Mel spectrum to obtain the MFCCs. This step decorrelates the Mel frequency bands and compresses the information into fewer coefficients.

(4) $$MFCC(j)=\sum_{i=1}^{M}\;L(i)\times cos(\frac{\pi j(2i-1)}{2M})\;$$

where MFCC(j) is the jth MFCC, M is the number of Mel filters, and L(i) is the log Mel spectrum from the previous step. Typically, the first 12–13 MFCCs are used as they contain the most relevant information about the spectral shape. Higher order MFCCs represent fast changes in the signal and are often discarded. In this work M = 32 Mel filter banks were used and retained the first 13 MFCC coefficients after applying the Discrete Cosine Transform (DCT).

### 2.4. 1D-CNN Augmented Transformer

In this study, we utilize a model that leverages the power of transformer architectures, specifically for sequences with temporal dependencies, and designate it as our novel You Only Listen Once (YOLO) model for automated prominent bowel sound detection. The model design employs Conv1D for initial feature extraction and subsequent layers of transformer blocks for more sophisticated processing. This section provides an analytical breakdown of each component in the model.

The model starts with a Conv1D layer for initial feature extraction from the sequence data. Given an input sequence x, the convolution operation is expressed as

(5) y(t)=x(t) ∗ w(t)

where * denotes the convolution operation, and w(t) represents the kernel or filter. This layer helps in extracting local patterns from the sequence. The output of this layer undergoes layer normalization and ReLU activation. Following the convolution layer, the model employs a series of transformer blocks, each comprising multi-head self-attention mechanisms and feed-forward networks. Given a sequence input, the multi-head self-attention mechanism computes the attention weights by considering three aspects: query Q, key K, and value V. The attention score for a given query–key pair is calculated as

(6) $$score(Q,\;K)=\frac{QK^{T}}{\sqrt{d_{k}}}$$

where d_k_ is the dimension of the key vectors. This score is then passed through a softmax function to produce the attention weights:

(7)  Attention(Q, K, V) = softmax(score(Q,K)) × V

The multi-head attention mechanism employs multiple such attention operations in parallel, with each operation working on different learned linear projections of the original Q, K, and V. Each transformer block also consists of a feed-forward network, which is applied to the output of the multi-head attention mechanism. This network can be represented by

(8) $$FFN(x)=max(0,\;xW_{1}+b_{1})W_{2}+b_{2}\;$$

where W1, W2, b1, and b2 are the weights and biases of the feed-forward network.

Each of these components in the transformer block is followed by dropout and layer normalization. After passing through the transformer blocks, the sequence undergoes global average pooling, effectively reducing its dimensionality by averaging the sequence elements.

The output from the pooling layer is then processed by fully connected layers (or dense layers). These layers are responsible for final feature transformation and integration. Given input x to a dense layer, the transformation can be represented by

(9) y=a(Wx+b)

where W is the weight matrix, b is the bias vector, and a is the ReLU activation function. Finally, the last dense layer has a sigmoid activation function, producing the model’s output. In summary, this model combines the benefits of convolutional layers for local feature extraction and transformer blocks for capturing long-range dependencies in sequence data, making it suitable for a wide range of sequential processing tasks. Table 1 highlights important parameters used in the 1D CNN–transformer architecture.

### 2.5. Training

Our dataset was divided into training, validation, and test sets (60/20/20% split). The model was conditioned to undergo a maximum of 150 epochs with a batch size of 512. Due to the stipulations set by the early-stopping mechanism, there existed the potential for training cessation prior to the full completion of the set epochs. The ModelCheckpoint callback was set to monitor and save the model at various training intervals, saving when a superior performance was detected on the validation set. Additionally, a mechanism to mitigate potential overfitting was introduced via the EarlyStopping callback. This function attentively observed the validation loss, ceasing training if no improvement was identified across 10 consecutive epochs.

Metrics such as loss, validation loss, accuracy, AUC, precision, and recall for each epoch were extracted and cataloged within the metrics_dict. This record was subsequently saved as a NumPy array, ensuring the availability of a comprehensive training history for future analyses without necessitating model retraining. Finally, the entirety of the trained model, encompassing both its architecture and the final weights, was serialized and saved for future testing.

## 3. Results

Figure 1 shows the arrangement of a device at two auscultatory areas. The captures from the top left and bottom right areas were sequential and not simultaneous.

**Figure 2:**
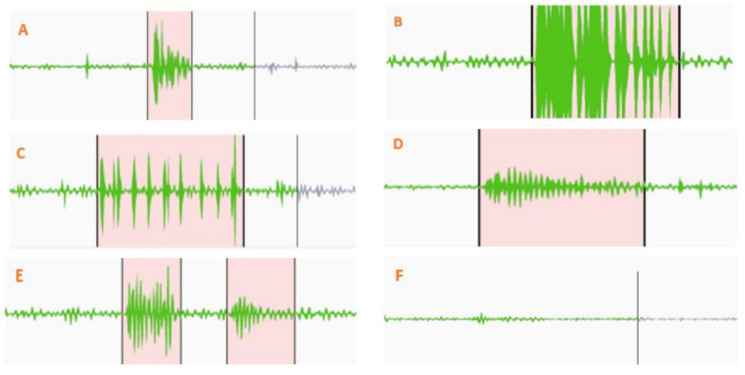
Annotated segments from bowel sound recordings. Panels (**A**–**D**) illustrate various types of clear prominent bowel sounds. Panel (**E**) shows the labeling of two closely occurring sounds, while panel (**F**) demonstrates a consistent, low-amplitude sound between distinct events, used as a marker for prominent sound differentiation.

**Figure 3:**
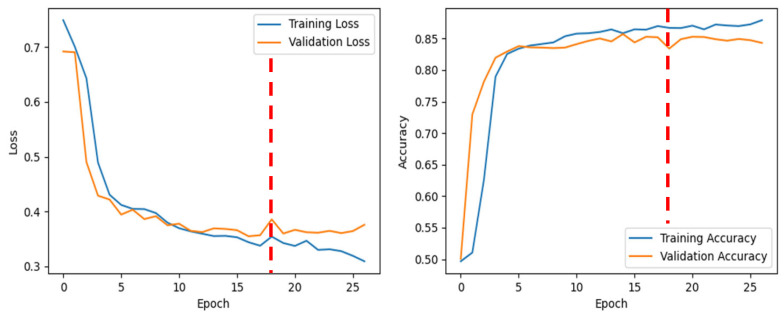
Training and validation performance across epochs.

Figure 2 shows various marked points from the bowel sound recordings, distinguishing between clearer and fainter sounds. Different types of clear bowel sounds can be seen in Figure 2A–D. Figure 2E demonstrates how two closely occurring sounds were labeled. A consistent baseline resting bowel sound between two distinct prominent bowel sounds served as a marker for differentiation. An example of this consistent sound is shown in Figure 2F. Figure 3 shows the training and validation performance of the model across epochs. The red dashed vertical line at epoch 18 marks the point at which early stopping was triggered during training. As seen from Figure 3, validation loss decreases consistently up to ~epoch 17, after which it plateaus and begins to increase slightly. Validation accuracy also reaches a near-maximal value around epoch 17–18 and then shows marginal variation without significant improvement.

We experimented with various segment sizes and batch sizes. Specifically we tested segment sizes of 0.5 s, 0.375 s, 0.25 s, and 0.125 s and batch sizes of 256, 512, and 1024. The optimal results were obtained with 0.375 s window data and a batch size of 512 exhibiting the highest accuracy and AUC among all configurations, as shown in Table 2. The model performance metrics were good, with accuracy 86.6%, precision 86.85%, recall 86.08, and F1 score of 0.864.

**Figure 4:**
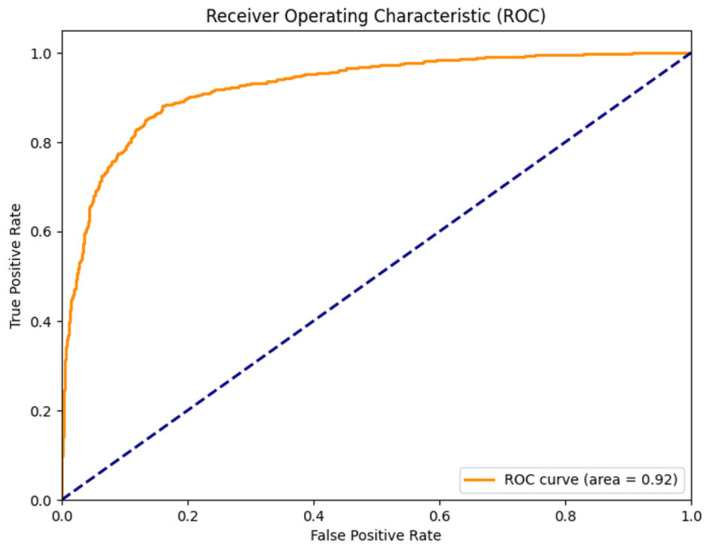
Receiver operating characteristic (ROC) curve for bowel sound classification (AUC = 0.92).

**Figure 5:**
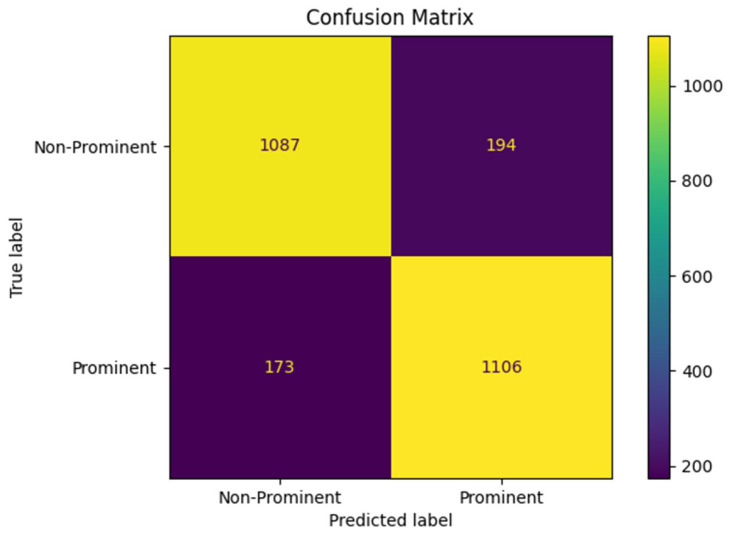
Confusion matrix for prominent vs. non-prominent bowel sound classification.

Figure 4 shows the receiver operating characteristic (ROC) curve for bowel sound classification with an AUC = 0.92. Figure 5 shows the confusion matrix for prominent vs. non-prominent bowel sound classification from the holdout test set. These results confirm the efficacy of the YOLO model, demonstrating its potential for real-time application towards digital transformation of gastroenterology practice and seamless integration into clinical applications.

## 4. Discussion

In this study, we aimed to develop a robust YOLO model for the detection of bowel sounds, which are vital indicators of gastrointestinal health. Our approach involved a comprehensive pipeline of data preprocessing, feature extraction, model development, training, optimization, and evaluation.

There have been previous studies which have taken into consideration different kinds of algorithms like FTT, moving average [22], perceptron [23], machine learning [24], SVM [25], CNN [26], Bayesian classifier [27], and CNN + RNN [28] detection of intestinal sounds by analyzing spectrograms using convolutional and recursive neural networks.

The main limitation of the approach includes the use of only a single sensor for recordings, the absence of data regarding the gastrointestinal response to an initial meal, and the unavailability of wireless functionality [28]. To ensure the quality of our input data, we loaded audio data using the librosa library and applied a high-pass Butterworth filter with a cutoff frequency of 50 Hz to eliminate low-frequency noise, which is common in physiological recordings. Normalizing the audio data and transforming them into a 16-bit integer format helped maintain consistency and facilitate further processing.

Liu J. [26] and colleagues developed a bowel sound detector using LSTM, capable of identifying the start and end points of each bowel sound by analyzing segments of the recordings. While they achieved high accuracy and sensitivity over 90% in their tests, the sensitivity notably decreased to 62% under actual usage conditions. This can be compared to another study, which had a good accuracy in detecting bowel sound types SB, MB, and HS [17], but lacked a comprehensive dataset. In our study, segmentation of the audio into 375 ms segments with a 200 ms overlap allowed us to capture the temporal dynamics of bowel sounds effectively. We categorized these segments based on the presence of prominent bowel sounds, providing labeled data for model training. Specifically, we trained the model using a dataset consisting of 6435 prominent sound segments and 36,149 non-prominent segments.

Feature extraction played a crucial role in capturing relevant information from the audio segments. We calculated Mel-frequency cepstral coefficients (MFCCs) using librosa, which are widely used in speech and audio processing tasks. Additionally, we applied Short-Time Fourier Transform (STFT) and Mel filter bank techniques to convert the time domain signals into the frequency domain, focusing on the most discriminative MFCCs for our model.

For model development, we adopted a 1D-CNN augmented transformer architecture, which combines the strengths of convolutional neural networks (CNNs) for feature extraction and transformers for capturing temporal dependencies in sequential data. The model comprised Conv1D layers for initial feature extraction, followed by transformer blocks with multi-head self-attention mechanisms and feed-forward networks. Global average pooling and fully connected layers were utilized for further processing, with the final layer employing a sigmoid activation function for binary classification.

During the training phase, we configured the model to train for up to 150 epochs with a batch size of 512, employing early stopping mechanisms based on validation loss to prevent overfitting. ModelCheckpoint and EarlyStopping callbacks were utilized to save the best-performing model based on validation set performance.

Optimization experiments were conducted to identify the optimal segment size and batch size for model training. We experimented with various segment sizes (0.5 s, 0.375 s, 0.25 s, and 0.125 s) and batch sizes (256, 512, and 1024) to find the optimal configuration, ultimately determining that a segment size of 0.375 s and a batch size of 512 yielded the best results in terms of accuracy and area under the curve (AUC) metrics.

The evaluation of the trained model demonstrated high performance in detecting bowel sounds, with an AUC of 0.93, accuracy of 86.6%, precision of 86.85%, and recall of 86.08%. These results indicate the effectiveness of our approach in accurately identifying prominent bowel sounds, which can have significant implications for gastrointestinal health monitoring and diagnosis.

A study utilized microelectromechanical system (MEMS) microphone-based sensor heads to record bowel sounds in a controlled, quiet environment. A limitation noted in the study was the system’s inability to perform effectively in noisy clinical settings, which could impact its practical application in hospitals or clinics where background noise is prevalent [29]. The outcomes of our research endeavor bear significant implications for both clinical practice and scientific exploration within gastroenterology, particularly in the domain of motility disorders. Our study endeavors to pioneer a sophisticated deep learning model capable of discerning and categorizing bowel sounds in real time, presenting an objective and efficient avenue for monitoring and diagnosing digestive ailments. Health professionals stand to benefit greatly from the integration of this system into their clinical armamentarium, facilitating expedited treatments, enhanced patient outcomes, and elevated standards of care.

The identification and classification of bowel sounds serve as pivotal initial steps in diagnosing a spectrum of gastrointestinal motility disorders, ranging from irritable bowel syndrome (IBS) to gastroparesis [30]. Early detection of these conditions holds the promise of timely interventions, potentially mitigating symptoms and enhancing patients’ overall quality of life [31,32].

Moreover, the integration of this system into telemedicine platforms and remote monitoring frameworks could democratize access to diagnostic capabilities, particularly benefiting underserved populations by enabling early detection of gastrointestinal anomalies. Beyond its immediate clinical implications, the methodological framework and findings of our study contribute substantively to the burgeoning field of artificial intelligence (AI) applications in gastroenterology, propelling advancements in digital healthcare technologies and fostering the emergence of personalized treatment modalities. In essence, our study underscores the transformative potential of deep learning systems in reshaping the landscape of gastroenterological care delivery.

Despite the considerable strides made in our investigation, it is essential to acknowledge the inherent limitations. The study’s reliance on a modest sample size comprising healthy adult volunteers may curtail the generalizability of findings to larger and more diverse populations, particularly those afflicted with gastrointestinal pathologies or comorbidities. Moreover, the data collection methodology primarily targeted specific anatomical regions, potentially overlooking the breadth and complexity of bowel sounds across diverse gastrointestinal segments.

Despite diligent efforts to mitigate noise and artifacts during data acquisition, environmental variables and individual idiosyncrasies in bowel sounds may introduce confounding factors. Additionally, the efficacy of the deep learning model may be susceptible to fluctuations in audio recording quality, variations in bowel sound characteristics, and the presence of extraneous physiological sounds or interferences.

While our model demonstrated commendable accuracy and efficiency in detecting bowel sounds under controlled conditions, its performance may exhibit variance in real-world scenarios characterized by diverse patient demographics and clinical contexts. Continual refinement and validation efforts are imperative to bolster the robustness and reliability of the deep learning system, thereby fortifying its utility in clinical diagnostic and therapeutic settings. Further research with a broader cohort of healthy and affected individuals is advocated to validate and generalize the findings, highlighting its potential in digital gastroenterology practice using phonoenterography [33]. Current work focuses on annotation-free adaptive YOLO model development to detect and characterize different prominent bowel sounds, estimate bowel rate (BR), bowel rate variability (BRV), and other metrics that can aid in the detection, diagnosis, and treatment monitoring of various bowel diseases.

## 5. Conclusions

In this research, we successfully demonstrated the potential of a novel You Only Listen Once (YOLO) deep learning model to effectively detect prominent bowel sounds in real time using the Eko DUO stethoscope. Leveraging a 1D-CNN augmented transformer with Mel-frequency cepstral coefficients, we achieved commendable performance metrics, signifying the model’s capability to classify bowel sounds. The optimal model configuration utilized a 0.375 s segment size and a batch size of 512. The insights obtained underscore the promising application of automated bowel sound detection in advancing digital gastroenterology. Future work should focus on a more diverse dataset, encompassing both healthy and pathologically afflicted subjects, to validate and generalize the findings.

## Figures and Tables

**Table 1:** Configuration of model architecture and training parameters for the YOLO-inspired CNN–Transformer.

Component	Parameter	Value/Setting
Input features	MFCCs per segment	13 coefficients × ~30 frames
	Segment duration	375 ms
	Sampling rate	44.1 kHz
Conv1D layer	Kernel size	3
	Number of filters	64
	Activation	ReLU
Transformer encoder	Number of encoder blocks	4
	Number of attention heads	8
	Embedding dimension	128
	Feed-forward layer size	256
	Dropout rate	0.1
Pooling layer	Type	Global average pooling
Dense layers	Hidden units	64
	Output activation	Sigmoid (binary classification)
Optimizer	Type	Adam
	Initial learning rate	0.001
Learning rate schedule	Scheduler	ReduceLROnPlateau
Loss function		Binary cross-entropy
Batch size		512

**Table 2:** Bowel sound classification performance across segment and batch sizes. Best performance (accuracy = 0.866, AUC = 0.930, F1 = 0.864) is highlighted in bold.

Segment Size (s)	Batch Size	Accuracy	AUC	Precision	Recall	F1 Score
0.5	256	0.847	0.922	0.867	0.818	0.841
0.5	512	0.848	0.921	0.847	0.851	0.833
0.375	256	0.856	0.929	0.838	0.883	0.859
0.375	512	**0.866**	**0.930**	**0.869**	**0.8608**	**0.864**
0.25	256	0.851	0.919	0.854	0.8481	0.851
0.25	512	0.855	0.919	0.8362	0.884	0.859
0.125	512	0.815	0.884	0.8138	0.821	0.817
0.125	1024	0.818	0.887	0.8264	0.8059	0.816

## Data Availability

Data used in this study are not for public use under the IRB protocol due to privacy and ethical restrictions.
